# The Physiology of Simultaneous Ambidextral Work

**Published:** 1904-03-26

**Authors:** 


### Hospital Clinics and Medical Progress.

### THE PHYSIOLOGY OF SIMULTANEOUS AMBIDEXTRAL WORK.

A lecture on this subject was delivered last week before the Ambidextral Culture Society by the Secretary, Mr. John Jackson. The lecturer referred to the equal skill in rapid and complicated movements of the two hands, which is displayed by skilled pianists, as a familiar example of simultaneous ambidextral skill. Indeed, among ordinary individuals who have culivated the use of only one hand there is, not merely a strong inclination to exercise both hands in totally different and unrelated duties which demand distinct and dissimilar movements at the same instant, but the exigencies of life and the imperative claims of certain occupations necessitate a very high standard of simultaneous skill, which standard there is no great difficulty in securing. But to the ambidextrous individual this synchronising development is natural and inevitable. The principles which it is the object of the Ambidextral Culture Society to maintain are:—(1) That it is possible to do two things well at the same time, and that every ordinarily intelligent person is capable of becoming as expert in the performance of two concurrent and unrelated acts as he can be in the separate accomplishment of one. (2) That the acts which are thus designated as concurrent or simultaneous are, so far as the senses can determine, absolutely so, whatever the refinement of analysis and science may ultimately declare them to be. For all practical purposes they occur precisely at the same instant, though they may theoretically be nothing more than inconceivably rapid alternations of volition. (3) That a certain amount of simultaneous work, under specified conditions, is healthy and expedient. (4) That under other particular conditions it is necessary and harmless. (5) That, whilst it is both proper and advantageous to prepare and qualify children for the execution of two-handed work, and more especially of two-handed writing, during their school life, it would be unwise, if not pernicious, to encourage or prescribe for artisans and others the least amount of simultaneous manual labour over and above what has hitherto been found requisite for the effective execution of the work to be done in the several industries of manufacturing life.

Sixty years ago Dr. A. L. Wigan published his monograph on "The Duality of the Mind," the expression of a belief 30 years old (the doctor himself was 60 when the book was published), which belief he says he had held all that time "with daily increasing conviction," and, further, he states that he "discussed the subject with more than a hundred of the first men in Europe," and that he "cannot remember a single objection made by a man of that stamp which has not vanished after my explanation." Among Dr. Wigan's 20 propositions are the following:—

"1. That each cerebrum is a distinct and perfect whole as an organ of thought.

"2. That a separate and distinct process of thinking or ratiocination may be carried on in each cerebrum simultaneously.

"3. That each cerebrum is capable of a distinct and separate volition, and that there are very often opposing volitions. . . .

"20. That every man is, in his own person, conscious of two volitions, quite distinct from the government of the passions by the intellect; a consciousness so universal that it enters into all figurative language on the moral feelings and sentiments, has enlisted into the service of every religion and forms the basis of some of them—as the Manichean."

In recent years Brown-Sequard and others have directed attention to the same question. The former states that "We have a great many motor elements in our brain which we neglect absolutely to educate. Such is the case particularly with the elements which serve for the movements of the left hand. Perhaps fathers and mothers will be more ready to develop the natural powers of the left hand of a child, giving it thereby two powerful hands, if they believe, as I do, that the condition of the brain and spinal cord would improve if all their motor and sensitive elements were fully exercised."

Apart from the opinions of individual authorities, corroborative evidence is to be found in familiar experience. How many clerks, for example, may be seen accurately casting up long rows of figures, while not merely conversing with another, but often telling an amusing tale with great rapidity, and this without an interruption or an interval of even a second of time in the narrative? In other pursuits similar evidence of concurrent mental processes can be adduced.

With regard to the question of whether the volitional impulses which govern the processes performed simultaneously by the two hands are really themselves simultaneous, or whether the "inconceivably rapid alternation" theory is correct, it is impossible to say, but the proposition is a correct one that these transmissions of will are progressing concurrently from two totally different and independent agencies, each of which is equal to its confrère in every particular so far as reason and the closest observation can see.

It is the aim and purpose of the Ambidextral Society to insure that every child at school shall be so drilled in both separate and simultaneous use of his two hands that whatever line of life he may enter he shall be fully prepared with two perfectly developed hands, equally sensitive, equally strong, and equally skilful. Such a scheme, whenever it is made an integral part of the national education, will undoubtedly raise the standard of excellence, and increase materially the effective working power of every individual, and of the community.

Illustrations of simultaneous ambidextral work were given on blackboards by Miss Daisy Jackson and several young ladies from the North Hackney

High School; the former drawing, at the request of one of the audience, a clock with the left hand, and the time indicated by the hands with the right hand, and the latter giving specimens of two-handed sumultaneous drawing in duplicate.

